# P-2134. Cryptococcosis in kidney transplant recipients: a single centre experience

**DOI:** 10.1093/ofid/ofaf695.2298

**Published:** 2026-01-11

**Authors:** Diego Oquendo-Gaona, Jhongert Alza-Arcila, Isabel Ramirez-Sanchez

**Affiliations:** Universidad de Antioquia, Medellin, Antioquia, Colombia; Universidad de Antioquia, Medellin, Antioquia, Colombia; Universidad de Antioquia, Hospital Pablo Tobon Uribe, Medellin, Antioquia, Colombia

## Abstract

**Background:**

Kidney transplant (KT) recipients have the lowest risk of Cryptococcosis among the solid organ transplant population, although this infection carries significant morbidity and mortality.

**Figure:**
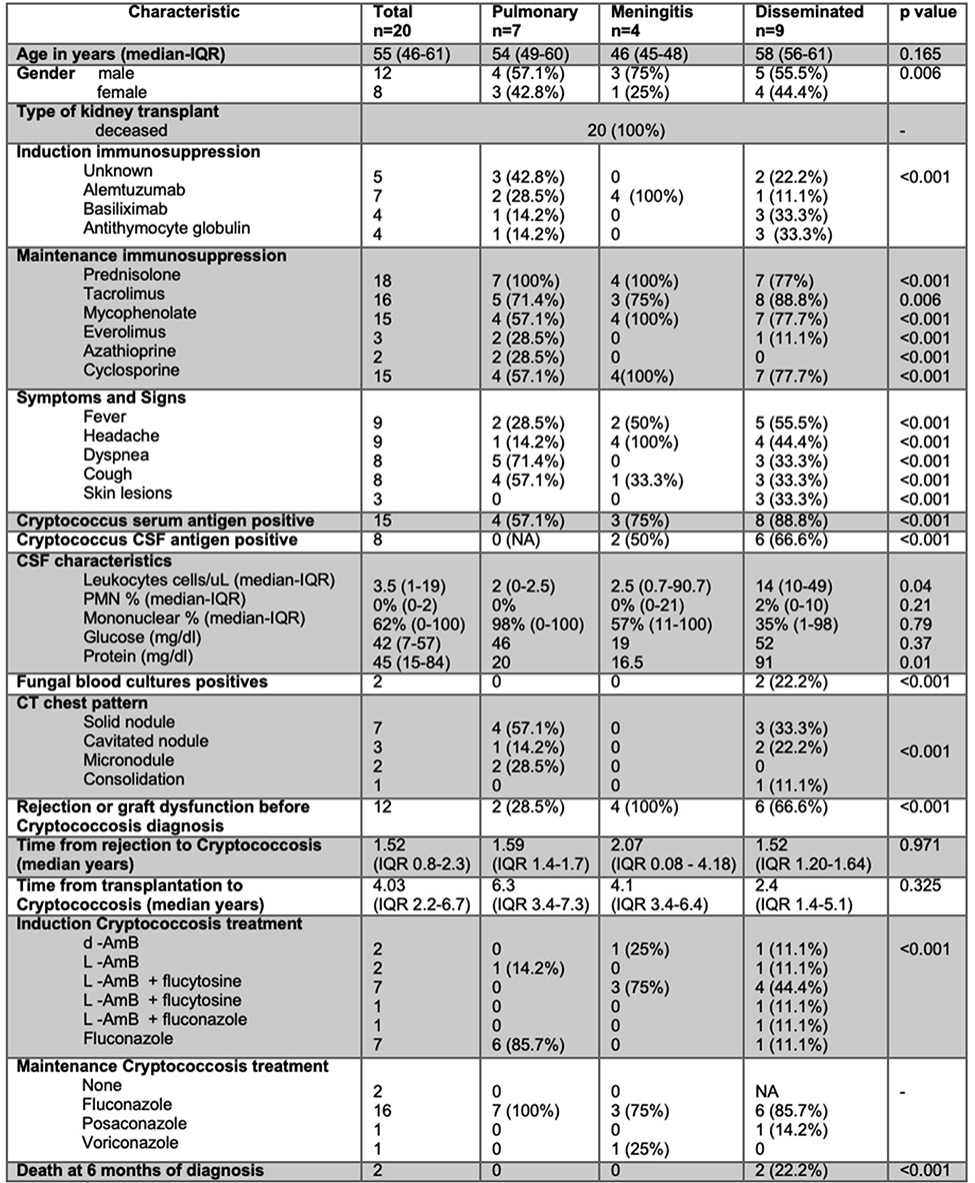
Demographic characteristics of Cryptococcosis in kidney transplant recipients

**Figure:**
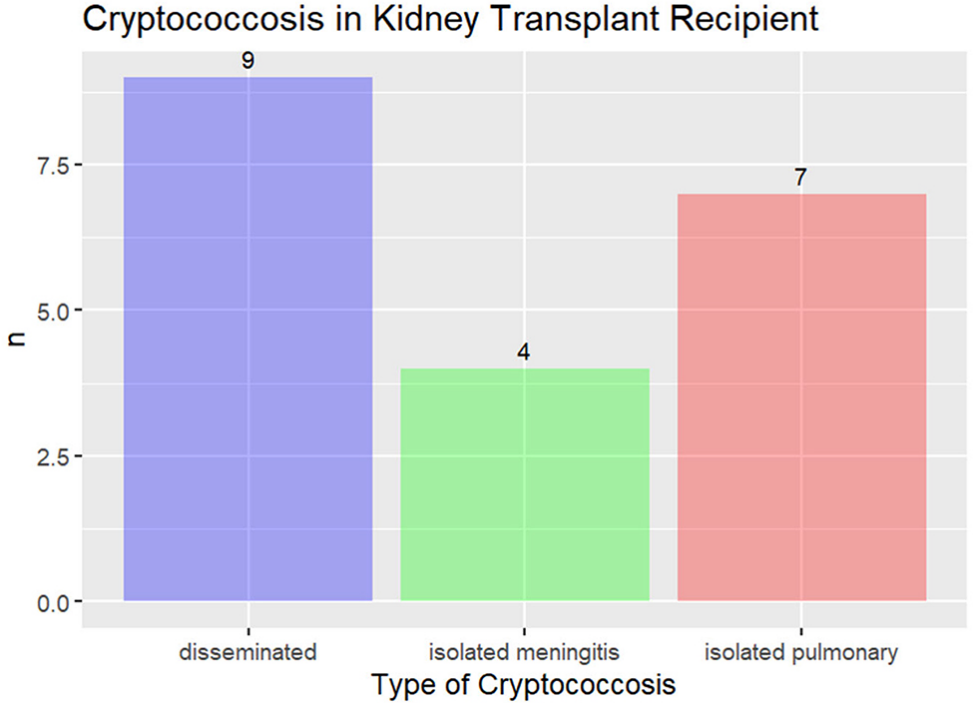
Distribution of Cryptococcosis clinical presentation among KT recipients

**Methods:**

Retrospective historical cohort in a single center in Medellin Colombia to analyse *C. neoformans* cryptococcosis in KT recipients from 2011 to 2024. Statistical analysis included chi-square tests for categorical variables, Kruskal-Wallis for continuous variables, and Kaplan-Meier survival analysis with log-rank tests.

**Figure:**
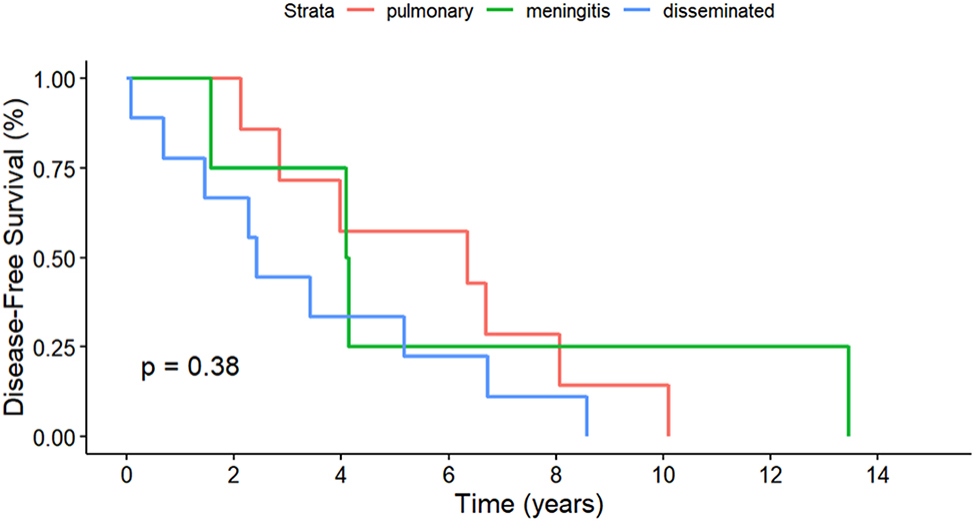
Kaplan-Meier Curve of cumulative % disease-free over time (years) in different forms of cryptococcosis in KT recipients

**Results:**

Twenty cases of cryptococcosis were diagnosed during the study period. Demographic data among KT recipients with isolated pulmonary cryptococcosis, isolated meningitis, and disseminated cryptococcosis showed comparable median ages (54, 46, and 58 years, respectively; p = 0.165), with male predominance in the meningitis and disseminated groups (75 and 55.5% respectively, p = 0.006). Median time from KT to infection was 4.03 years (IQR 2.2-6.7) and median time from rejection to cryptococcosis was 1.52 years (IQR 0.8-2.3). The most common clinical manifestations at diagnosis were: fever (28.5–5.5%, p < 0.001), headache (100% in meningitis/disseminated, p < 0.001), and respiratory symptoms mainly in pulmonary cases. Increasing positivity for serum Cryptococcal antigen from pulmonary cases (57.1%) to disseminated cases (88.8%, p < 0.001). Cerebrospinal fluid analysis showed mild pleocytosis (14 cells/µL) and hyperproteinorrachia in meningitis/disseminated cases. Chest CT revealed distinct radiological patterns, with solid nodules in pulmonary (57.1%) and disseminated (33.3%) disease. Amphotericin B plus flucytosine was the therapy of choice in meningitis/disseminated disease, whereas fluconazole was used primarily for mild pulmonary disease, followed by fluconazole maintenance therapy. Six-month deaths occurred exclusively in disseminated cases (22.2%, p < 0.001) and disease-free survival showed no significant difference between groups (p = 0.38).

**Conclusion:**

In KT recipients, the clinical manifestations of Cryptococcosis are varied. Although disease-free survival was similar between groups, disseminated disease carries the highest risk of death, highlighting early detection and targeted therapy to achieve the best outcome.

**Disclosures:**

All Authors: No reported disclosures

